# Comparison of different monitoring methods for the measurement of metaldehyde in surface waters

**DOI:** 10.1007/s10661-019-7221-x

**Published:** 2019-01-15

**Authors:** Glenn D. Castle, Graham A. Mills, Anthony Gravell, Alister Leggatt, Jeff Stubbs, Richard Davis, Gary R. Fones

**Affiliations:** 1 School of Earth and Environmental Sciences, University of Portsmouth, Burnaby Road, Portsmouth, PO1 3QL UK; 2 School of Pharmacy and Biomedical Sciences, University of Portsmouth, White Swan Road, Portsmouth, PO1 2DT UK; 3 Natural Resources Wales, NRW Analytical Services, Swansea University, Faraday Building, Singleton Campus, Swansea, SA2 8PP UK; 4 Affinity Water Ltd., Tamblin Way, Hatfield, Hertfordshire, AL10 9EZ UK; 5 Anatune Ltd, Unit 4, Wellbrook Court, Girton Road, Cambridge, CB3 0NA UK

**Keywords:** Metaldehyde, Water monitoring, Drinking water, Spot sampling, Passive sampling, On-line gas chromatography/mass spectrometry

## Abstract

Metaldehyde is recognised as an emerging contaminant. It is a powerful molluscicide and is the active compound in many types of slug pellets used for the protection of crops. The application of pellets to land generally takes place between August and December when slugs thrive. Due to its high use and physico-chemical properties, metaldehyde can be present in the aquatic environment at concentrations above the EU Drinking Water Directive limit of 100 ng L^−1^ for a single pesticide. Such high concentrations are problematic when these waters are used in the production of drinking water. Being able to effectively monitor this pollutant of concern is important. We compared four different monitoring techniques (spot and automated bottle sampling, on-line gas chromatography/mass spectrometry (GC/MS) and passive sampling) to estimate the concentration of metaldehyde. Trials were undertaken in the Mimmshall Brook catchment (Hertfordshire, UK) and in a feed in a drinking water treatment plant for differing periods between 17th October and 31st December 2017. This period coincided with the agricultural application of metaldehyde. Overall, there was a good agreement between the concentrations measured by the four techniques, each providing complementary information. The highest resolution data was obtained using the on-line GC/MS. During the study, there was a large exceedance (500 ng L^−1^) of metaldehyde that entered the treatment plant; but this was not related to rainfall in the area. Each monitoring method had its own advantages and disadvantages for monitoring investigations, particularly in terms of cost and turn-a-round time of data.

**Electronic supplementary material:**

The online version of this article (10.1007/s10661-019-7221-x) contains supplementary material, which is available to authorized users.

## Introduction

Metaldehyde (C_8_H_16_O_4_) is now considered an emerging pollutant of concern. It is a cyclic tetramer of acetaldehyde and is used as potent molluscicide. Metaldehyde is the active compound in many propriety types of slug bait in use worldwide (Bieri 2003). It is used agriculturally to protect a wide range of crops, including oil seed rape, wheat and winter barley from unwanted pests (Simms et al. 2006). It is most frequently used in the autumn and winter when slugs and snails tend to thrive in the wetter environment (Green 1996). Between 2008 and 2014, it was estimated that in Great Britain arable farmers used ~ 1640 t of pellets containing metaldehyde (FERA 2018). Metaldehyde is polar (log *K*_ow_ = 0.12 at 20 °C), soluble in water (0.188 g L^−1^ at 20 °C) and mobile in soil (PPDB 2018). After application to land, during wet conditions, it can run-off into field drains and surface waters (Kay and Grayson 2014). Issues relating to metaldehyde in the environment have been reviewed (Castle et al. 2017).

Levels of metaldehyde found in environmental waters fluctuate with seasonal application of the molluscicide. High usage of metaldehyde has led to frequent detections in surface waters above the EU Drinking Water Directive (DWD) limit of 0.1 μg L^−1^ for any single pesticide. In the UK water industry, this is referred to as the prescribed concentration value (PCV)) (European Commission 1998). There is a potential risk when these waters are used subsequently for potable supplies (Drinking Water Inspectorate 2017). Further issues arise as removing metaldehyde from contaminated supplies can be difficult. For example, this compound is hard to remove when using conventional granular activated carbon beds as water treatment processes (Busquets et al. 2014). More specialised treatment techniques e.g. ultra violet radiation and oxidation processes can be used to remove metaldehyde; these processes require high capital investment and are expensive to operative (Castle et al. 2017). Alternative application approaches (e.g. subsidising the use ferric phosphate as an alternative molluscicide) and river catchment management plans have been developed to help to reduce metaldehyde concentrations in surface waters within the UK. For example, the Metaldehyde Stewardship Group has created the “Get pellet-wise” initiative with the aim to work with farmers on the timing and application rates of metaldehyde (Metaldehyde Stewardship Group 2018). In order to gauge the performance of these remedial actions and initiatives, a viable water monitoring programme for metaldehyde needs to be established. Such programmes need to take into consideration the sporadic presence of this pollutant due to the stochastic nature of the inputs that are also linked to rainfall events and other environmental factors (e.g. soil type, soil saturation index and slope) within a given river catchment. Hence, ideally, the monitoring method used should be responsive and able to provide information in a timely fashion to end-users so as to enable them to mitigate for any environmental risks.

A number of different water quality methods are available for monitoring a pollutant like metaldehyde in surface water, each with their associated advantages and disadvantages. The most common procedure approach is spot (bottle or grab) sampling that involves the periodic removal of a small volume of water for subsequent analysis at a remote laboratory. This procedure is routinely applied by water supply companies as part of their regulatory monitoring programmes. The method is low-cost, but has some limitations (Gong et al. 2018). For example, collected samples often require pre-concentration prior to analysis, and this can be time consuming; the concentrations obtained can be misrepresentative especially where there are sporadic inputs of pollutants into the aquatic environment and the response time is slow (Rabiet et al. 2010). One way of increasing the resolution temporally is by increasing the frequency of spot sampling or using automated water collection systems (e.g. time or event triggered bottle samplers). The use of automated samplers has some disadvantages in that they are expensive to purchase, require regular maintenance and can be used only at relatively secure field sites. Additionally, the increased number of samples collected during a monitoring programme adds significantly to the operating costs of the analytical laboratory. The use of on-line telemetric sensors that can be linked to a remote control centre to enable management decisions (e.g. cessation of abstracting water going into a treatment works) provides the highest degree of temporal resolution and responsiveness with the ability to catch and react to stochastic pollution events. Although some sensor-based systems have been proposed for the measurement of metaldehyde (e.g. Lonestar™ portable detection system, that utilises a field asymmetric ion mobility spectrometer (Castle et al. 2017), none are in routine use as an effective monitoring tool at the intake of drinking water treatment plants.

An alternative approach to water quality monitoring overcoming many issues associated with spot sampling is the use of passive samplers. These devices have been introduced as a method for providing more representative (e.g. time-weighted average [TWA]) concentrations of pollutants in water (Townsend et al. 2018; Castle et al. 2018a). Passive samplers offer many advantages including low-cost, are non-mechanical, requiring no external energy source and can be deployed in a wide range of different field situations. A number of different devices have been developed to monitor different types of organic pollutants occuring in surface waters (Vrana et al. 2005). These samplers include semi-permeable membrane devices, polymer sheets (e.g. low-density polyethylene or silicone rubber) or Chemcatcher® for non-polar pollutants (Lohmann et al. 2012) and the polar organic chemical integrative sampler (POCIS) (Van Metre et al. 2017; Alvarez et al. 2004), o-DGT (Guibal et al. 2017; Challis et al. 2016; Chen et al. 2013) or the polar version of the Chemcatcher® (Petrie et al. 2016) for polar pollutants. For the measurement of polar analytes, samplers are comprised of an inert body that houses the receiving phase that is selective for the analytes of concern. Normally, the receiving phase is overlaid by a thin diffusion membrane. Samplers can be deployed for varying amounts of time (e.g. 7–28 days) where compounds are sequestered continually from the environmental medium. The measurement of the TWA concentration of a pollutant requires the compound-specific sampler uptake rate (*R*_*s*_, normally expressed as the equivalent volume of water cleared per unit time (L day^−1^)) needed (Vrana et al. 2005). *R*_*s*_ can be measured using either, laboratory or in situ field calibration experiments (Castle et al. 2018b). Mathematical models based on the physicochemical properties of a chemical can also be used to predict *R*_*s*_ (Challis et al. 2016; Miller et al. 2016; Booij et al. 2007). Recently, a bespoke Chemcatcher® passive sampler suitable for monitoring metaldehyde in surface waters has been developed (Castle et al. 2018b). The sampler comprises an inert PTFE body containing a hydrophilic-lipophilic-balanced Horizon Atlantic™ HLB-L disk as receiving phase, overlaid with a thin polyethersulfone (PES) diffusion membrane (Castle et al. 2018b).

This study aimed to investigate a number of different monitoring approaches for the measurement of metaldehyde in surface water and in an influent stream entering a drinking water treatment plant. The monitoring was undertaken during the period when metaldehyde was being applied to land within the river catchment. This was likely to result in sporadic inputs of the molluscicide into surface water. Four different methods were evaluated including spot water sampling, automated bottle sampling, on-line gas chromatography/mass spectrometry (GC/MS) system and passive sampling. Their performance was evaluated in terms of their ability to provide robust and representative concentrations of metaldehyde which could be used subsequently in environmental risk assessments and to facilitate better management of water abstraction and also reduce the risk of regulatory exceedances.

## Materials and methods

### Monitoring site

The trial was undertaken at Mimmshall Brook, which is situated in Hertfordshire, Southern England. This river catchment area is primarily arable farmland (20.8 km^2^) growing oil seed rape (3.12 km^2^), winter wheat and other cereals (11.5 km^2^). Both metaldehyde and ferric phosphate are used in this area agriculturally to control mollusc infestations. Part of the brook flows into a large karstic swallow hole system where it mixes with groundwater. The resultant water in the swallow holes is heavily influenced by the quality of the surface water. This mixed water source is abstracted (9.09 ML day^−1^) by Affinity Water Ltd., the local drinking water supply company. This source together with three others are used as potable supplies (31.5 ML day^−1^) supplying a large population within Hertfordshire and North London. Over the past 8 years, concentrations of metaldehyde above the PCV have been detected frequently in this water that supplies the drinking water treatment plant. This presents an operational risk for the company. Inside the plant, the supply water from groundwater influenced by the swallow hole network is first clarified to reduce turbidity and then passed over granular activated carbon beds (for removal of organic chemicals), followed by membrane ultra-filtration and finally disinfection.

### Monitoring at Mimmshall Brook

Three different monitoring techniques (spot sampling, automated bottle sampling and passive sampling) were trialled at Mimmshall Brook between 17th October and 14th November 2017. This corresponded to the agricultural application period of metaldehyde in the river catchment. Over the trial, the water temperature in the Brook varied between 8.0 and 12.5 °C.

#### Spot water sampling

Over the trial, two independent sets of spot water samples were collected by the University of Portsmouth (weekly duplicates) and Affinity Water Ltd. (five samples collected during their routine water monitoring programme). Spot samples of water gathered in this study followed methods described by Castle et al. (2018a). Briefly, samples were collected into either plastic bottles (250 mL) (University of Portsmouth) or amber screw top glass bottles (40 mL) containing sodium thiosulphate solution (0.36% *w*/*v*, 0.25 mL) as preservative (Affinity Water Ltd.). All samples were stored at ~ 4 °C until analysis, undertaken within 14 days of collection. Under these storage conditions, there was no measurable loss of analyte. Metaldehyde was quantified in the spot water samples (University of Portsmouth) by liquid chromatography tandem mass spectrometry (LC-MS/MS). The instrument (Agilent 1200RR LC system coupled to an Agilent 6460 tandem mass spectrometer (Agilent Technologies, Santa Clara, USA)) was interfaced with an on-line solid-phase extraction system containing a Waters Oasis® HLB cartridge. The method limit of quantification (LoQ) was 10.0 ng L^−1^, defined as three times the limit of detection. This procedure has been described in full by Schumacher et al. (2016).

The Affinity Water Ltd. spot samples were analysed in their nationally accredited (United Kingdom Accreditation Service, UKAS) laboratory using a routine and validated electrospray ionisation LC-MS/MS (Agilent 6490) method (ISO/IEC 17025:2005) for the quantification of metaldehyde in water (Castle et al. 2018a). An on-line solid-phase extraction system connected to the liquid chromatograph was used for sample analysis. The mobile phase was a 0.1% acetic acid:acetonitrile gradient. Samples were spiked with internal standard (metaldehyde-d_16_, > 99 atom % deuterium) and sodium thiosulphate added before analysis. The MS/MS was operated in the multiple reaction mode, with the sodiated adduct ion for metaldehyde monitored by the first quadrupole (Castle et al. 2018a). LoQ was 9.0 ng L^−1^, defined as three times the limit of detection.

#### Automated bottle sampling

A HACH portable automated bottle sampler (model AS950, https://www.hach.com/as950-peristaltic-samplers/portable-samplers/family?productCategoryId=35547137070) was used to collect daily (sampler triggered at 09.00 h each day) water samples (250 mL) over the trial period as part of the Affinity Water Ltd. routine monitoring programme. During the same working day, the water sample was removed and then decanted into an amber screw top glass bottles (40 mL) containing sodium thiosulphate solution (0.36% *w*/*v*, 0.25 mL). Samples were stored at ~ 4 °C (for up to 14 days after collection) and analysed for metaldehyde by Affinity Water Ltd. using the analytical procedure as described previously.

#### Chemcatcher® passive samplers

The preparation and processing of the Chemcatcher® samplers has been described previously by Castle et al. (2018b). Briefly, PTFE Atlantic design Chemcatcher® bodies (Fig. S1) (AT Engineering, Tadley, UK) were soaked overnight (5% Decon 90 solution) (Decon Laboratories Ltd., Hove, UK), washed in water and acetone and finally rinsed in water and dried. The receiving phase was a Horizon Atlantic™ hydrophilic-lipophilic balanced (HLB-L) disk (47 mm diameter) (Labmedics Ltd., Abingdon, UK) and activated by passing (under a gentle vacuum) HPLC grade methanol (50 mL) then HPLC grade water (100 mL) through the disk. In order to prevent the disks from drying out, after activation, they were left in Milli-Q water. The overlying PES diffusion membrane (Supor® 200, 0.2 μm pore diameter; cut to 52 mm diameter disks) (Pall Europe Ltd., Portsmouth, UK) was cleaned by soaking (12 h) in methanol, washed in water and kept damp until use. Devices were assembled by placing a HLB-L receiving phase disk onto the sampler supporting base plate followed by a PES membrane. Finally, the sampler components were secured in place using the Chemcatcher® retaining ring. Samplers were kept immersed in Milli-Q water until use. Before field use, a small quantity of water was added to the top well and the sampler lid fitted and secured tightly.

Two devices were deployed (using a robust plastic sheet, (Fig. S2), ensuring that the samplers remained submerged) for consecutive periods of 2 weeks. A field blank was exposed at deployment and retrieval. It was then resealed and processed as for the field deployed samplers. Earlier work in our laboratory showed that the Chemcatcher® was in the time integrative (linear) uptake mode for in excess of 2 weeks for metaldehyde, thus allowing TWA concentrations to be calculated (Castle et al. 2018b). This also limited biofouling of the PES membrane. After each field deployment, samplers were sealed using the lid, transported to the laboratory in a cool box and maintained at ~ 4 °C until analysed (usually within 1 week).

Exposed Chemcatcher® samplers were dissembled, and the HLB-L receiving phase disk dried (48 h at room temperature) on methanol-rinsed aluminium foil. The PES membranes were discarded. Each HLB-L disk was eluted (methanol, 40 mL) using a glass extraction funnel manifold (under gravity). The eluent was collected into glass vials (60 mL). In order to prevent losses of metaldehyde, water (1 mL) was added to the vial to act as a keeper). The solution was evaporated (~ 0.5 mL) using a Genevac ‘Rocket’ centrifugal rotary evaporator (Genevac Ltd., Ipswich, UK). Afterwards, the extract was transferred to a vial (2 mL) and the volume adjusted to 1 mL with methanol. Metaldehyde in these extracts was analysed as for the spot water samples (University of Portsmouth method) with the following modification. The extract (100 μL) was added to a silanised glass auto-sampler vial containing water (900 μL) and labelled internal standard solution (20 μL of metaldehyde-d_16_, 50 μg L^−1^) and then analysed as previously. The method LoQ was 0.45 ng L^−1^, defined as three times the limit of detection. This LoQ is lower (~ 20) than that achieved by the procedure used for the analysis of the spot water samples. Effectively, over the 14-day deployment period, the Chemcatcher® samples 224 mL of water and therefore this accounts for the improved LoQ.

The TWA concentration of metaldehyde measured by the Chemcatcher® was calculated using Eq. 1.

1 $$ Cw=\frac{M_{S(t)}-{M}_0}{R_S\times t} $$

where:

- *C*_*w*_ *=* concentration (ng L^−1^) of analyte in water.
- *M*_*S(t)*_ = mass (ng) of analyte in Chemcatcher® receiving phase disk after exposure time *t* (day).
- *M*_*0*_ = mass (ng) of analyte in receiving phase disk of Chemcatcher® field blank.
- *R*_*S*_ = sampler uptake rate of analyte (L day^−1^).

In a previous laboratory calibration study, *R*_*s*_ was determined as 16 mL day^−1^ (Castle et al. 2018b). This uptake rate was measured at a water velocity of ~ 0.2 m s^−1^ over the face of the sampler bodies and a water temperature of (5.0 ± 1.0 °C). These conditions were selected as they correspond to the flow velocity and water temperature of rivers in the UK during the late autumn to winter months when metaldehyde is most likely to be present in impacted catchments.

### Monitoring in the plant at post-clarifier feed

Three different monitoring techniques (spot water sampling, on-line GC/MS system and passive sampling) were trialled in the post-clarifier feed of the drinking water treatment plant coinciding with the agriculturally application of metaldehyde in the river catchment.

#### Spot water sampling

Two sets of spot water samples were collected by University of Portsmouth (duplicate weekly samples between 17th October and 14th November 2017) and Affinity Water Ltd. (16 single samples collected between 20th October and 28th December 2017). The collected spot samples of water were analysed for metaldehyde using the two analytical procedures as described previously.

#### Chemcatcher® passive samplers

Chemcatcher® passive samplers were prepared as above. Duplicate samplers were deployed (17th October–14th November 2017) for consecutive periods of either 7 days, 14 days or 28 days in a bespoke stainless steel sink enclosure (AT Engineering, Tadley, UK) (Fig. S3) capable of holding up to six devices on two circular plates. Samplers were attached, using cable ties, faced down to stainless steel plates. Water from the post-clarifier feed of the drinking water treatment plant was piped into the enclosure at a flow rate of ~ 5.5 L min^−1^, and this allowed an upwelling of the water that then overflowed to waste. This design permitted the samplers to be continuously exposed to the test water over the trial. The water temperature over the trial varied between 11.0 and 13.5 °C. After each deployment period, samplers were removed and handled and analysed for metaldehyde using the procedures as described previously. A blank device was exposed during each deployment and retrieval operation, and after resealing was processed as for the exposed samplers in the tank.

#### On-line gas chromatography/mass spectrometry system

Since September 2016, an on-line GC/MS system has been installed at the Affinity Water Ltd. drinking water treatment plant. This bespoke system analyses three different streams within the plant including the post-clarifier feed. The system was installed so as to provide rapid, high-frequency data on the concentration of metaldehyde in the water entering and leaving the granular-activated carbon bed. The approach was to take an existing validated and accredited laboratory-based GC/MS method (Maury 2012) for the analysis of metaldehyde and to transfer this into a robust, dedicated on-line system at the drinking water treatment plant. The GC/MS system comprised an Agilent 7890A gas chromatograph (fitted with a GERSTEL cooled injection system) connected to an Agilent 7000 triple quadrupole mass spectrometer. Prior to analysis, water samples were filtered (to reduce turbidity < 1 NTU) and passed through a controllable flow cell (1 L min^−1^) (Ridgway 2014) (Fig. S4). Samples were extracted using a GERSTEL MPS 2 XT dual head device fitted with a pre-conditioned solid-phase cartridge (20 mg ISOLUTE® ENV+ sorbent, Biotage). This hyper-crosslinked hydroxylated polystyrene-divinylbenzene copolymer sorbent has a high surface area and is highly retentive of polar analytes. The water sample (7.5 mL) together with labelled metaldehyde-d_16_ internal standard (1 mL) was loaded onto the cartridge and allowed to dry for 15 min using a nitrogen flow. This ensured a high recovery of metaldehyde. After drying, the sample was eluted (into a 2-mL GC vial) using dichloromethane (400 μL) and then injected (10 μL) directly onto the GC/MS instrument. Metaldehyde was quantified using multiple reaction monitoring. The limit of detection of the method was 3 ng L^−1^. Analysis of each stream took approximately 1 h. Quality control samples were extracted and run daily to ensure satisfactory operating performance. Data was transmitted telemetrically control centre, but it was not linked directly to control plant processes. The whole system was contained in a purpose-built laboratory grade, air conditioned cabin to maintain correct operating and environmental conditions. Further details of the methods are provided elsewhere (Davis et al. 2017).

## Results and discussion

### Comparison of monitoring methods at Mimmshall Brook

**Fig. 1:**
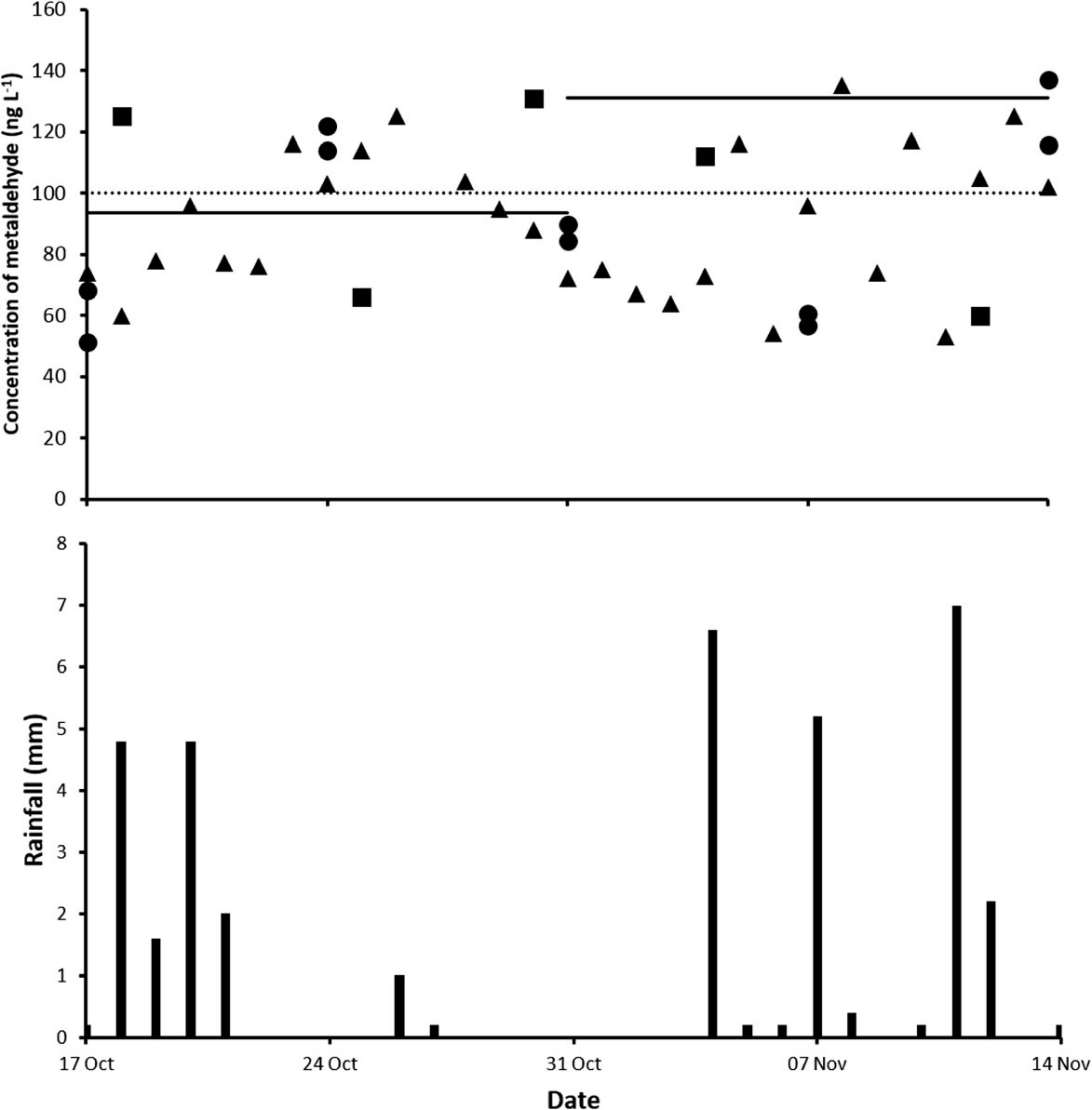
Concentration of metaldehyde (ng L^−1^) at Mimmshall Brook measured (University of Portsmouth (●), Affinity Water Ltd. (■) and automated bottle sampler (▲)) in spot samples of water, together with time-weighted average (TWA) concentrations measured using the Chemcatcher® (^**_____**^) between 17 October and 14 November, 2017. The line (∙∙∙∙∙∙∙) shows the European Union’s Drinking Water Directive limit of 100 ng L^−1^ for a single pesticide. LoQ for spot samples of water was 10 ng L^−1^ (University of Portsmouth) and 9 ng L^−1^ (Affinity Water Ltd.) and for the Chemcatcher® extracts was 0.45 ng L^−1^. Local daily rainfall (mm) was measured at the Environment Agency weather station (ID 276316TP)

The concentrations of metaldehyde in spot samples of water and with the automated bottle sampler over the 4-week trial are shown in Fig. 1 and Table 1. Metaldehyde was quantifiable in all samples collected, and there were frequent marginal exceedances of the European Union’s Drinking Water Directive limit of 100 ng L^−1^ for a single pesticide (European Commission 1998). There was agreement between the two monitoring methods with the concentration of metaldehyde varying over the trial between 51 and 137 ng L^−1^. There was evidence that concentrations in the Brook changed on a sub-daily basis, indicating highly sporadic inputs of the molluscicide. Rainfall in the area over this period varied between 0 and 7 mm (Fig. 1). There was a slight association between periods of higher rainfall in weeks 3 and 4 and elevated concentrations of metaldehyde in the Brook. Linking concentrations of metaldehyde found in surface water to rainfall directly is problematic as there are several additional influential factors that need to be considered (Asfaw et al. 2018; Castle et al. 2017). Factors include method and application rates of metaldehyde, croppage, field slope and drainage, soil type and soil moisture deficit (Lu et al. 2017).

**Table 1:** Mean concentration (± standard deviation) and range of metaldehyde (ng L^−1^) at Mimmshall Brook measured (17 October–14 November, 2017) using two spot water sampling procedures and automated bottle sampler and time-weighted average (TWA) concentrations measured by the Chemcatcher®. *n* = number of samples

Monitoring method	Weeks 1–2	Weeks 3–4
University of Portsmouth spot water samples	88 ± 24 range = 51–122 *n* = 6	91 ± 29 range = 56–137 *n* = 6
Affinity Water Ltd. spot water samples	107 ± 29 range = 66–131 *n* = 3	86 ± 26 range = 60–112 *n* = 2
Combined spot water samples	95 ± 28 range = 51–131 *n* = 9	89 ± 28 range = 57–137 *n* = 8
Automated bottle sampler	91 ± 18 range = 60–125 *n* = 15	89 ± 26 range = 53–135 *n* = 15
Combined spot water and automated bottle sampler samples	93 ± 22 range = 51–131 *n* = 24	84 ± 27 range = 53–137 *n* = 23
Chemcatcher® 1	93	147
Chemcatcher® 2	95	115
Chemcatcher® average	94	131

The TWA concentrations of metaldehyde measured using the Chemcatcher® are given in Fig. 1 and Table 1. Metaldehyde detected in exposed field blank devices was below the LoQ (< 0.45 ng L^−1^). There was no visual evidence of biofouling of the PES membrane over the 14-day deployments. The average TWA concentration was higher during weeks 3–4 (131 ng L^−1^) compared with weeks 1–2 (94 ng L^−1^). For the first deployment, there was good agreement between the mean values and the average TWA concentrations measured by the different monitoring methods (Table 1). There was less agreement for the second deployment; however, the average TWA concentration still fell within the range (56–137 ng L^−1^) found with the University of Portsmouth spot water sampling method. However, there is no data on the variation of the concentration of metaldehyde in the Brook during the periods when spot samples of water were not collected. Overall, it can be considered that both approaches gave similar results and hence can be used effectively to monitor metaldehyde in the aquatic environment. These findings agree with Castle et al. (2018a and 2018b) who also found that the Chemcatcher® gave complementary data to that obtained using spot water sampling methods.

### Monitoring in the plant at the post-clarifier feed

#### Comparison of on-line GC/MS with spot sampling methods

**Fig. 2:**
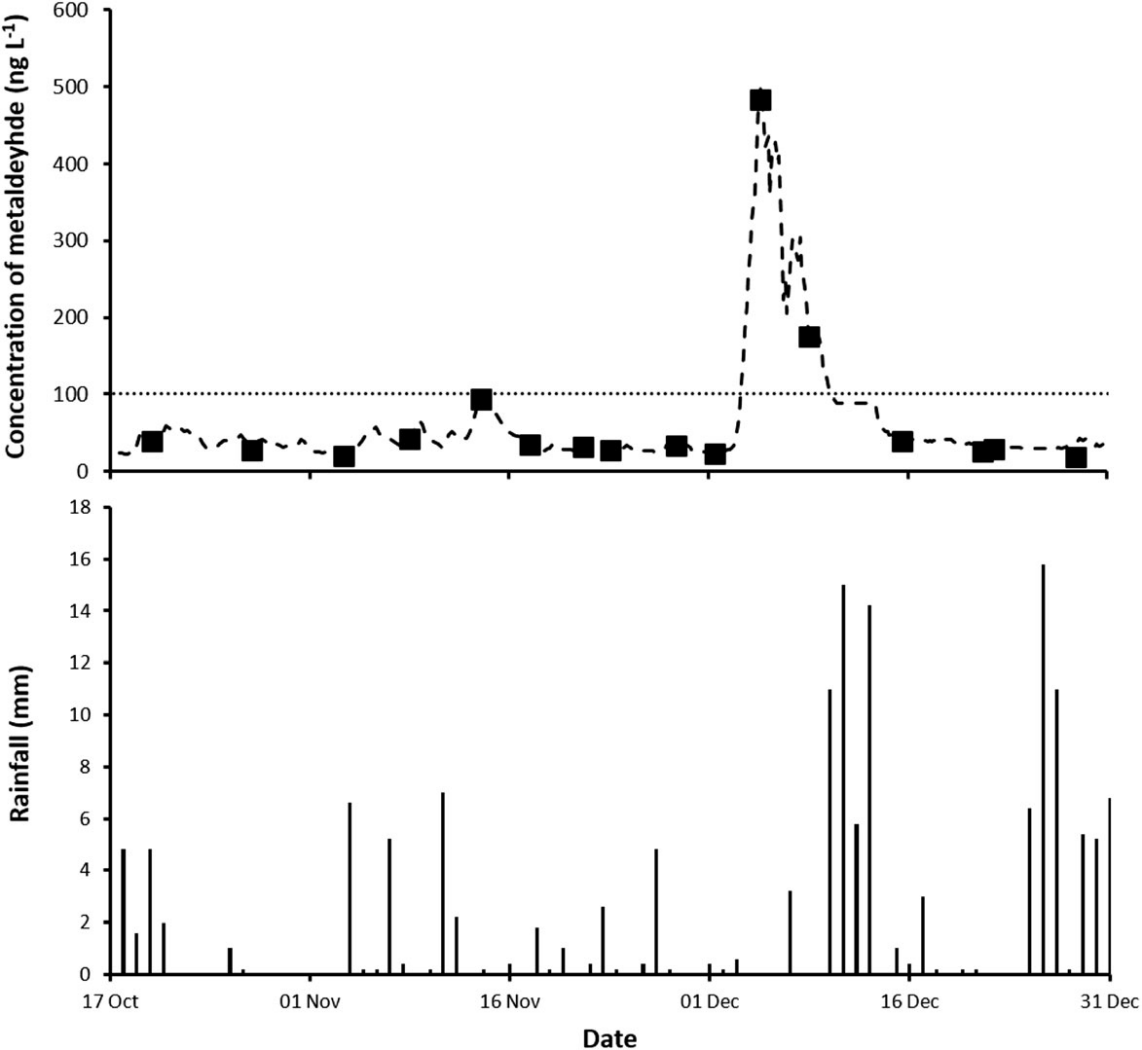
Concentration of metaldehyde (ng L^−1^) measured in the plant post clarifier feed with spot samples of water (Affinity Water Ltd. (■)) and the on-line GC/MS system (− − −) between 17 October and 31 December, 2017. The line (∙∙∙∙∙∙∙) shows the European Union’s Drinking Water Directive limit of 100 ng L^−1^ for a single pesticide. LoQ for spot samples of water was 9 ng L^−1^ (Affinity Water Ltd.) and the limit of detection for the on-line GC/MS system was 3 ng L^−1^. Local daily rainfall (mm) was measured at the Environment Agency weather station (ID 276316TP)

Concentration data from the on-line GC/MS channel was obtained at a frequency of approximately every 3 h (giving ~ 600 values) and this is plotted for the trial period (17th October–31st December, 2017) in Fig. 2. Over this period, there were no values below the limit of detection (3 ng L^−1^). The novel on-line system was capable of operating automatically over extended periods giving rugged and robust high-frequency information on the variation of the concentration of metaldehyde. We are unaware of such an on-line system being in operation at a plant elsewhere. Between the 3rd-10th December, 2017, there was a sustained and elevated concentration (peaking at ~ 500 ng L^−1^) of metaldehyde that exceeded the European Union’s Drinking Water Directive limit for all of this time period. This concentration is at the limit for ‘total’ pesticides permissible in drinking water under the above Directive. This exceedance could have presented a potential risk to the operability of the drinking water treatment plant if the capacity of the granular activated carbon beds was inadequate to completely remove the continual high load of metaldehyde. At present, the on-line GC/MS instrument is not interfaced to a process control centre within the drinking water treatment plant where decisions on whether to continue to abstract from the source can be made remotely. Once this capability is enabled, this will represent a major change in the operability of the works, so that additional water treatment is only required when a pre-set trigger value is exceeded. This should help to extend the lifetime of the granular activated carbon beds and thereby reduce operational costs at the plant.

The concentrations of metaldehyde measured in 16 routine water samples collected during the trial period are shown in Fig. 2. There was good agreement between the two monitoring approaches, particularly considering both use different analytical methods (GC/MS or LC/MS) for determining metaldehyde. Fortunately, a spot sample was taken that coincided with the peak concentration of metaldehyde on 4th December, 2017, otherwise this serious pollution event could easily have been missed using this monitoring approach. This is a major drawback of the use of infrequent spot water sampling. As was found in the Mimmshall Brook study, there was no direct link between rainfall and increased concentrations of metaldehyde. The major exceedance occurred in a dry period with rainfall not above 0.6 mm. By early December, metaldehyde would have been applied agriculturally for the previous 4 months and this could have led to a build-up of pellets on the land.

#### Comparison of Chemcatcher® with on-line GC/MS and spot sampling methods

**Fig. 3:**
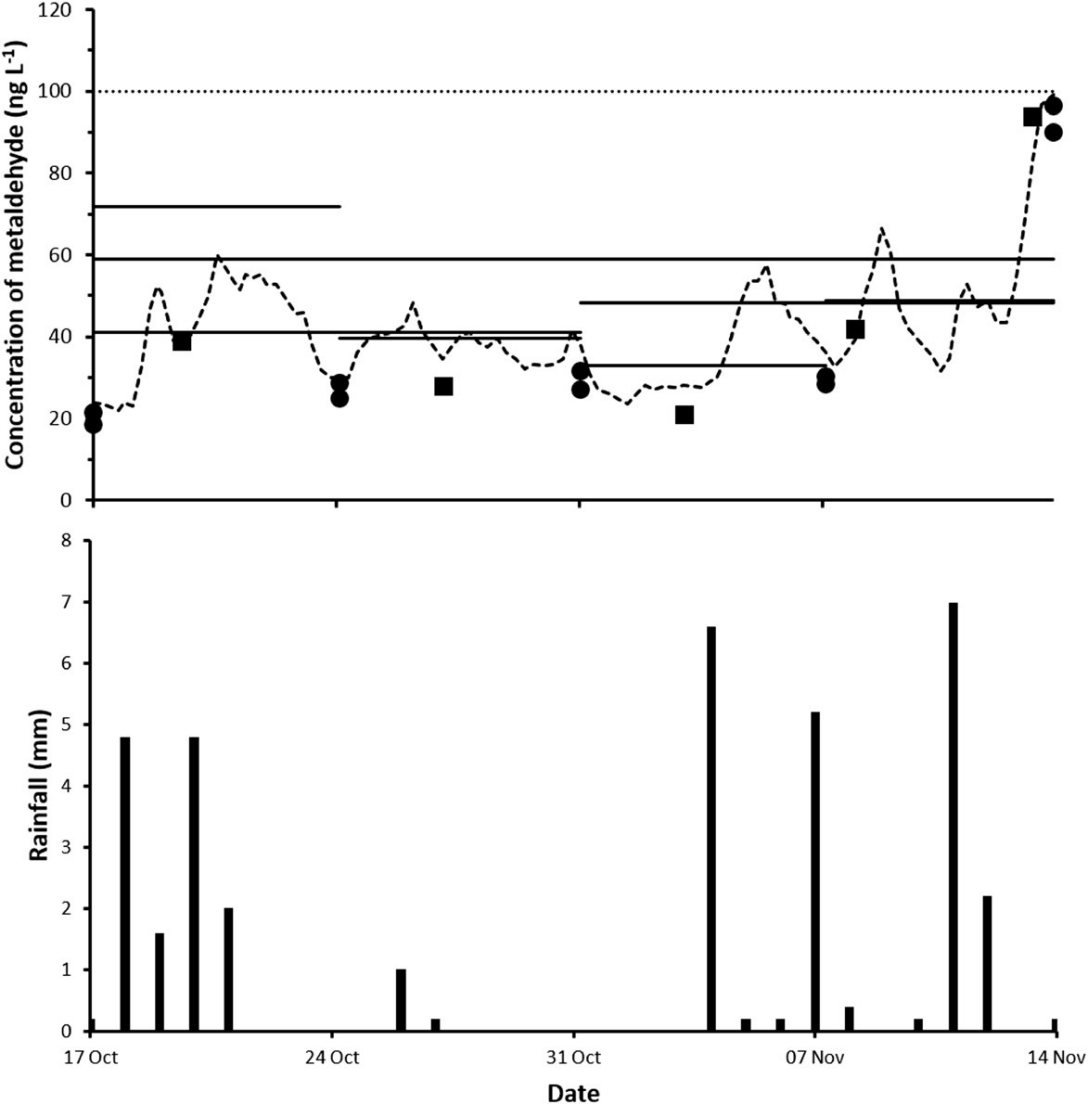
Concentration of metaldehyde (ng L^−1^) measured in the plant post clarifier feed with spot samples of water (University of Portsmouth (●) and Affinity Water Ltd. (■)) and the on-line GC/MS system (−−), together with time-weighted average (TWA) concentrations measured with the Chemcatcher® (^**____**^) between 17 October and 14 November, 2017. The line (∙∙∙∙∙∙∙) shows the European Union’s Drinking Water Directive limit of 100 ng L^−1^ for a single pesticide. LoQ for spot samples of water was 10 ng L^−1^ (University of Portsmouth) and for the Chemcatcher® extracts was 0.45 ng L^−1^. The limit of detection for the on-line GC/MS system was 3 ng L^−1^. Local daily rainfall (mm) was measured at the Environment Agency weather station (ID 276316TP)

TWA concentrations of metaldehyde measured during the different Chemcatcher® exposure periods together with the values obtained using the on-line GC/MS and spot sampling methods are shown in Fig. 3 and Table 2. Over this more limited trial period, there were no exceedances of the European Union’s Drinking Water Directive limit. As indicated previously, there was good agreement in the concentrations measured in both sets of spot water samples (Affinity Water Ltd. and University of Portsmouth) and the on-line GC/MS system. The mass of metaldehyde detected in exposed Chemcatcher® blank samplers was less than the LoQ (< 0.45 ng L^−1^). The PES membrane showed little visual evidence of biofouling over the varying deployment periods. Generally, there was good agreement with the TWA concentrations and the two other monitoring methods. A higher TWA concentration (72 ng L^−1^) was found in week 1 of the trial compared with the mean value (39 ± 13 ng L^−1^) obtained using the other techniques. The reason for this anomaly is unknown; however, a possible cause is that the PES membranes either moved within the PTFE sampler body during their preparation or storage or were damaged during this deployment. These issues would lead to a greater sequestration of metaldehyde similar to that observed previously for acidic herbicides (Townsend et al. 2018). There was good agreement in the TWA concentration obtained with each of the duplicate samplers for all of the trial periods, showing the reproducibility of the device. This is likely to be attributable to the immobilised sorbent in the form of a disk used as the receiving phase in the Chemcatcher® (Mills et al. 2014; Castle et al. 2018b). This second evaluative trial of the Chemcatcher® also shows how the device can provide comparable data with that obtained using either infrequent spot water sampling or high-frequency on-line monitoring methods.

**Table 2:** Mean concentration (± standard deviation) and range of metaldehyde (ng L^−1^) over the different Chemcatcher® exposure periods in the plant post clarifier feed measured (17 October–14 November, 2017) using two spot sampling procedures and the on-line GC/MS system, together with the time-weighted average (TWA) concentrations found in the Chemcatcher® passive sampler. *n* = number of samples

Monitoring method	Week 1	Week 2	Week 3	Week 4	Week 1–2	Week 3–4	Week 1–4
University of Portsmouth spot water samples	24 ± 4 range = 19–29 *n* = 4	28 ± 2 range = 25–32 *n* = 4	30 ± 2 range = 27–32 *n* = 4	61 ± 32 range = 28–97 *n* = 4	25 ± 4 range = 19–32 *n* = 6	51 ± 30 range = 27–97 *n* = 6	40 ± 27 range = 19–97 *n* = 10
Affinity Water Ltd. spot water samples	39 *n* = 1	28 *n* = 1	21 *n* = 1	68 ± 26 range = 42–94 *n* = 2	34 ± 6 range = 28–39 *n* = 2	52 ± 31 range = 21–94 *n* = 3	45 ± 26 range = 21–94 *n* = 5
Combined spot water samples	27 ± 7 range = 19–39 *n* = 5	28 ± 2 range = 25–32 *n* = 5	28 ± 4 range = 21–32 *n* = 5	64 ± 30 range = 28–97 *n* = 6	27 ± 6 range = 19–39 *n* = 8	51 ± 30 range = 21–97 *n* = 9	42 ± 27 range = 19–97 *n* = 15
On-line GC/MS	42 ± 12 range = 22–59 *n* = 30	38 ± 4 range = 30–48 *n* = 27	36 ± 10 range = 23–58 *n* = 29	52 ± 18 range = 32–99 *n* = 24	40 ± 9 range = 22–59 *n* = 56	43 ± 17 range = 23–99 *n* = 52	42 ± 14 range = 22–99 *n* = 107
Combined spot water and GC/MS samples	39 ± 13 range = 19–59 *n* = 35	36 ± 5 range = 25–48 *n* = 32	35 ± 10 range = 21–58 *n* = 34	54 ± 22 range = 28–99 *n* = 30	38 ± 10 range = 19–59 *n* = 64	44 ± 20 range = 21–99 *n* = 61	42 ± 16 range = 19–99 *n* = 122
Chemcatcher® 1	78	43	31	52	41	54	58
Chemcatcher® 2	65	36	34	46	41	43	59
Chemcatcher® average	72	40	33	49	41	48	59

### Effect of variation of Chemcatcher® uptake rate

Since the concentration of metaldehyde in the post-clarifier feed between 17 October and 14 November 2017 did not vary widely, the experiment provided an opportunity to estimate in-situ *R*_*s*_ values. This was undertaken by rearranging Eq. 1 and calculating the average concentration of metaldehyde (Table 2) together with the TWA concentration and the amount of metaldehyde sequestered on the HLB-L disk during the different exposure periods. The estimated in-situ *R*_*s*_ values are shown in Table 3. Previously, using the Chemcatcher® in a semi-static laboratory calibration experiment and an in-situ field calibration, the *R*_*s*_ value for metaldehyde was determined as 15.7 mL day^−1^ (water temperature = 5 ± 1 °C) and 17.8 mL day^−1^ (water temperature = 13–14 °C) respectively (Castle et al. 2018b). Apart from our week 1 exposures in the post-clarifier feed, the *R*_*s*_ values obtained were in general agreement with those found in the previous study. The best comparative *R*_*s*_ estimates (14–27 mL day^−1^) were found using the on-line GC/MS mean water concentrations for metaldehyde (Table 3) as this technique provided the highest number of data points. Some of this variation may be attributed to both differences in water temperature and likely differences in the water velocity over the face of the sampler bodies in the different studies and exposure periods. A higher water velocity would lead to greater turbulence, a reduced diffusive boundary layer and hence a higher sampling rate. Overall, this shows the robustness and reliability of the Chemcatcher®, and that *R*_*s*_ values for this polar pollutant did not vary widely with differing environmental conditions (Mutzner et al. 2018); this is in contrast to the sequestration of non-polar contaminants (Huckins et al. 2002). However, with the latter class of pollutants, performance reference compounds can be used to accommodate changes in both water temperature and water turbulence (Estoppey et al. 2016; Allan et al. 2009). Use of performance reference compounds with samplers designed to monitor polar chemicals has not shown to be effective (Harman et al. 2012).

**Table 3:** Time-weighted average (TWA) concentrations (ng L^−1^) and mass (ng) of metaldehyde on receiving phase disk using the Chemcatcher® passive sampler, deployed in the plant post clarifier feed between 17 October and 14 November, 2017. The sampler uptake rate (*R*_*s*_, mL day^−1^) was calculated (Eq. 1) using the different mean water concentrations (ng L^−1^) obtained over the varying deployment periods (for values see Table 2). The water temperature in the exposure tanks varied between 11.0 and 13.5 °C. The number of samples used to calculate *R*_*s*_ for the different deployments is shown in Table 2

	Week 1	Week 2	Week 3	Week 4	Week 1–2	Week 3–4	Week 1–4
TWA Chemcatcher® 1	78	43	31	52	41	54	58
TWA Chemcatcher® 2	65	36	34	46	41	43	59
TWA Chemcatcher® average	72	40	33	49	41	48	59
Mass on disk Chemcatcher® 1	8.6	4.7	3.5	5.7	9.1	11.8	25.6
Mass on disk Chemcatcher® 2	7.2	4.0	3.8	5.0	9.0	9.4	26.1
Mass on disk average	7.9	4.4	3.6	5.4	9.0	10.6	25.9
*R*_*s*_ calculated using University of Portsmouth mean spot water sample concentration	47	22	17	13	26	15	23
*R*_*s*_ calculated using Affinity Water Ltd. mean spot water sample concentration	29	22	25	11	19	15	21
*R*_*s*_ calculated using mean of combined spot water sample concentration	42	22	18	12	24	15	22
*R*_*s*_ calculated using mean of on-line GC/MS concentration	27	16	14	15	16	18	22
*R*_*s*_ calculated using mean of combined spot water and GC/MS concentration	29	17	15	14	17	17	22

## Conclusions

This paper has evaluated the suitability and reliability of four different monitoring methods for the quantitative measurement of metaldehyde. It has demonstrated some of the challenges of monitoring polar pollutants that are present in surface water only sporadically. Infrequent spot and automated bottle sampling methods and their associated analytical techniques have sufficient sensitivity (LoQ ~ 10 ng L^−1^) to detect metaldehyde in the aquatic environment. Using infrequent spot sampling, however, there is a high likelihood that regulatory exceedances can be missed. Hence, there is a need to continually blend with different supply sources less impacted by metaldehyde to ensure compliance with the current directives. The use of high frequency automated bottle monitoring can be used as an alternative approach; however, as we have shown, the concentration of metaldehyde can change on a sub-daily basis. Collecting, for example, hourly samples would add significantly to laboratory costs. With both off-line methods, there is also a time delay in obtaining results back from the analytical laboratory, and this will also impact on the operability of the drinking water treatment plant.

Use of the on-line GC/MS overcomes all of the limitations of these above techniques. The system can yield high quality data on the concentration of metaldehyde with approximately a 1-h turn-a-round time. The GC/MS measurements were reliable and in close agreement with those obtained by spot sampling. The main drawback of the monitoring method is high cost. However, this initial investment can be off-set over time by the reducing plant operating costs.

Passive sampling provides another cost-effective alternative for monitoring metaldehyde. Our field trials have shown that the Chemcatcher® provides TWA concentrations in broad agreement with both the spot, bottle and on-line methods. There was little variability in the estimated *R*_*s*_ value and, hence, this gives credibility of using the sampler in routine monitoring campaigns. A drawback is that passive samplers cannot yield information on the peak or maximum concentration that the sampler was exposed to during the deployment. Furthermore, passive samplers cannot provide rapid data as they are deployed typically for periods of 7–14 days. However, passive samplers can be used on the catchment scale to investigate sources and fluxes of this problematic molluscicide, especially at sites where water is being removed as a source for the production of potable supplies. If samplers are deployed at the intake of a drinking water treatment plant, they can be used together with water flow to estimate the mass loadings of a pollutant entering the works. These estimates can be used to better determine the operational lifetime of the granular activated carbon beds. Passive samplers can also provide information on the performance of remediation schemes (e.g. use of ferric phosphate as an alternative molluscicide).

## Electronic supplementary material

ESM 1 (DOCX 2225 kb)
