# Supplementary material for: Comparison of different monitoring methods for the measurement of metaldehyde in surface waters
Source: Environ Monit Assess. 2019 Jan 15;191(2):75. doi: 10.1007/s10661-019-7221-x (PMC6333724; doi:10.1007/s10661-019-7221-x)
Supplement: Supplementary file 1 — (DOCX 2225 kb) [file 10661_2019_7221_MOESM1_ESM.docx]

**Electronic supplementary material**

**Comparison of different monitoring methods for the measurement of metaldehyde in surface waters**

Glenn D. Castle^a^, Graham A. Mills^b^, Anthony Gravell^c^, Alister Leggatt^d^, Jeff Stubbs^e^, Richard Davis^e^ and Gary R. Fones^a*^

^a^School of Earth and Environmental Sciences, University of Portsmouth, Burnaby Road, Portsmouth, PO1 3QL, UK

^b^School of Pharmacy and Biomedical Sciences, University of Portsmouth, White Swan Road, Portsmouth, PO1 2DT, UK

^c^Natural Resources Wales, NRW Analytical Services, Faraday Building, Swansea University, Singleton Campus, Swansea, SA2 8PP, UK

^d^Affinity Water Ltd., Tamblin Way, Hatfield, Hertfordshire, AL10 9EZ, UK

^e^Anatune Ltd, Unit 4, Wellbrook Court, Girton Road, Cambridge, CB3 0NA, UK

^*^Corresponding author: email: [gary.fones@port.ac.uk](mailto:gary.fones@port.ac.uk); Tel.: 00 44 (0)2392 842252

***
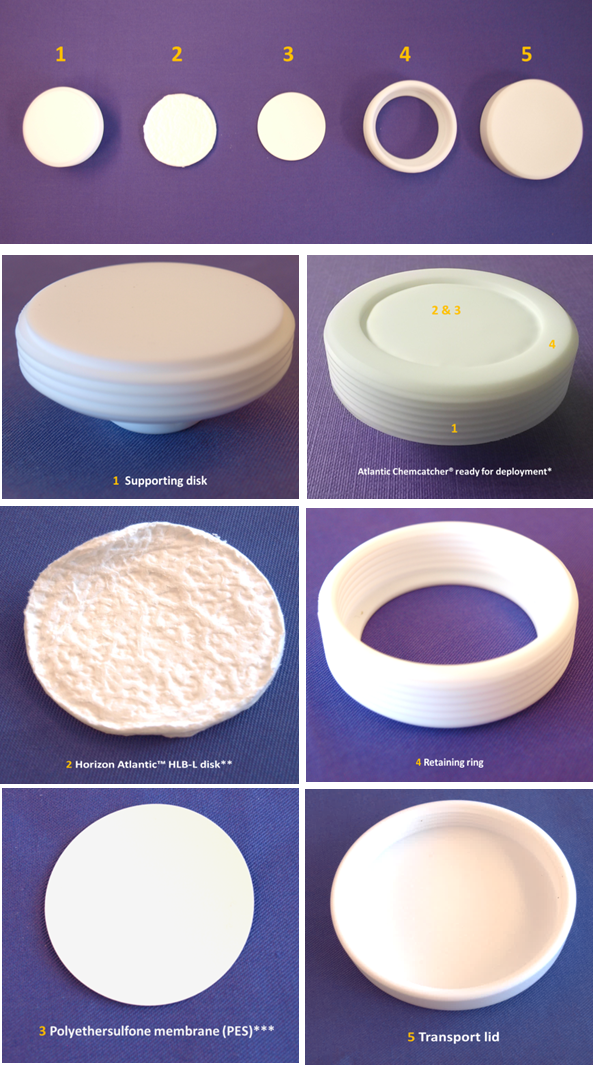
***

**Figure S1:** Components of the Horizon Atlantic® version of the Chemcatcher® passive sampling device. *The Chemcatcher® is made of machined PTFE, **Horizon Atlantic® HLB-L disk (47 mm diameter) and ***polyethersulfone membrane (PES), Supor®200, pore size: 0.2 µm, 52 mm diameter disk.

Buoy (flotation) device)

Carabiners

Water flow

Water level

Acrycast plastic sheet with two Chemcatcher® samplers, secured by split pins

Sheet secured to rope by cable ties

Paving slab 400 x 400 mm

Eye secured to paving slab with bolts and washers

Chemcatcher® samplers deployed ~ 0.25 m below water surface

**Figure S2**. Schematic of rig used to deploy duplicate Chemcatcher® passive samplers in Mimmshall Brook.


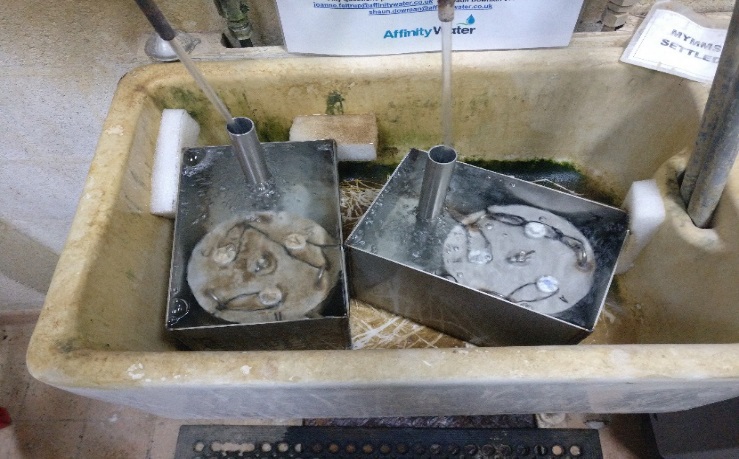


Post-clarifier feed tap.

Overflow to sink.

Stainless steel pipe allowing upwelling of water into the enclosure.

Upper stainless steel plate holding three Chemcatcher devices with cable ties. The equivalent lower stainless steel plate is not visible.

Stainless steel tank enclosure.

**Figure S3.** Stainless steel sink enclosure used to deploy Chemcatcher^®^ passive samplers at the post-clarifier inlet at the drinking water treatment plant.

**Figure S4.** GERSTEL dual head multi-purpose sampler with flow cell.
